# Efficacies of Various Anaerobic Starter Seeds for Biogas Production from Different Types of Wastewater

**DOI:** 10.1155/2017/2782850

**Published:** 2017-08-28

**Authors:** Pawinee Chaiprasert, Nasrul Hudayah, Chompoonut Auphimai

**Affiliations:** ^1^Biotechnology Program, School of Bioresources and Technology, King Mongkut's University of Technology Thonburi, Bangkhunthian, Bangkok 10150, Thailand; ^2^ECoWaste, The Joint Graduate School of Energy and Environment, King Mongkut's University of Technology Thonburi, Thungkru, Bangkok 10140, Thailand

## Abstract

Various anaerobic starter seeds from different sources were investigated for their efficacies in treatment of different types of wastewater. Six combinations of starter seeds and wastewaters were selected out of 25 combination batch experiments and operated in semicontinuous reactors. It was noticed that the efficacies of various anaerobic starter seeds for biogas production from different types of wastewater in terms of reactor performance and stability were depended on wastewater characteristics and F/M ratio affecting microbial community and their microbial activities. However, exogenous starter seed can be used across different types of wastewater with or without acclimatization. Four reactors reached the targeted OLR of 2 kg COD/m^3^·d with high performance and stability except for concentrated rubber wastewater (RBw), even using high active starter seeds of cassava starch (CSs) and palm oil (POs). The toxic compounds in RBw such as ammonia and sulfate might also adversely affect methanogenic activity in CSsRBw and POsRBw reactors. DGGE analysis showed that propionate utilizers,* Smithella propionica* strain LYP and* Syntrophus* sp., were detected in all samples. For* Archaea* domain, methylotrophic, hydrogenotrophic, and acetoclastic methanogens were also detected. Syntrophic relationships were assumed between propionate utilizers and methanogens as acetate/H2 producers and utilizers, respectively.

## 1. Introduction

Alternative energy generation options, which are economic well-being with green future, are demanded to accomplish global energy demand and environmental concerns [1]. Production of bioenergy has shown an impressive growth concerning energy demands with sustainable perspectives. Anaerobic digestion gains its popularity for organic wastewater treatment because it proves an excellent biological process for waste stabilization by both recovering on energy and compost. It also offers simple and compact technology with high COD removal efficiencies and low sludge production which lead to economical and feasible application in industrial scale of wastewater treatment. Methane, as a main product in anaerobic digestion, was able to generate approximately 1.5 kWh electric energy per kg COD removed with assumption of 40% electric conversion efficiency from methane [2]. Therefore, anaerobic digestion is considered as the most appropriate method to treat organic wastewaters, such as food processing and agricultural based industries.

In Thailand, wastewaters from agricultural based industries are main sources for biogas production [3]. These wastewaters had different characteristics which affected the operational design of anaerobic digestion system. Start-up strategy, one of operational parameters in anaerobic reactor, must be considered in order to operate anaerobic reactor with good stability for long period. Poor start-up can cause longer acclimation of microorganism to wastewater which then leads to inefficient organic removals or reactor fail [4]. Start-up of anaerobic reactor is commonly affected by several parameters such as reactor configuration, wastewater characteristics, environmental conditions, and microbial starter seeds [5]. Selection of appropriate microbial starter seeds is considered as critical factor to assure the good performance of anaerobic reactor [6, 7].

For starting up anaerobic reactor, anaerobic sludge or granule from another reactor is commonly used and inoculated to new anaerobic reactor for the reasons of its high microbial activity and congeniality [5, 8]. However, the limitation on its availability is a main drawback for starting up anaerobic reactor. Providing an alternative or exogenous starter seed which has good microbial activity and is easy or available nearby the wastewater treatment plant is considered. Selection for new starter seeds must be focused only not on the quantity of its source and amount, but also on the quality of it, such as microbial activity and community [5]. In this study, microbial starter seeds from 5 different wastewater treatment plants, for example, cassava starch (high carbohydrates), palm oil mill (high oil and grease), soymilk processing (high protein), concentrated rubber (high protein), and swine manure (high nitrogen and lignocellulosic matter) wastewater treatment plants, will be investigated for their suitability as exogenous starter seeds or inoculum. This study was aimed to investigate the efficacies of exogenous microbial starter seeds, in terms of microbial activity and performance, to treat different wastewater types. Therefore, the results of this study were expected to contribute on the knowledge and critical parameters for starting up new anaerobic reactor by using exogenous microbial starter seeds.

## 2. Materials and Methods

### 2.1. Starter Seeds

The starter seeds were collected from full-scale anaerobic reactors which were operated more than 5 years with normal organic loading rate and high efficiency (more than 75% COD removal) under ambient temperature of 30–35°C. Five anaerobic starter seeds used in this study were (1) rubber starter seed (RBs) which was collected from anaerobic wastewater treatment plant (WWTP) of concentrated rubber factory; (2) cassava starch seed (CSs) which was collected from anaerobic covered lagoon of WWTP at cassava starch factory; (3) palm oil starter seed (POs) which was collected from anaerobic WWTP treating POME at palm oil factory; (4) swine starter seed (SWs) which was collected from swine manure WWTP at local farm; (5) soymilk starter seed (SMs) which was collected from anaerobic pond WWTP at soy milk processing factory. These starter seeds were characterized for their biomass characteristics, for example, volatile suspended solids (VSS) and total suspended solids (TSS), specific glucose utilization (SGU), specific methanogenic activity (SMA), and microbial community.

### 2.2. Wastewater Characteristics

5 different wastewaters were also used in this study such as wastewater of concentrated rubber (RBw), cassava starch (CSw), palm oil mill effluent (POw), swine manure (SWw), and soymilk processing (SMw). These wastewaters were collected from the same factory as the starter seeds. The characteristics of these wastewaters are shown in Table 1.

### 2.3. Experimental Design

A factorial experiment was designed for two variables of starter seeds and wastewaters. 5 different starter seeds and wastewaters were carried out in a 5 × 5 factorial experiment design as shown in Table 2. These 25 experiments were conducted in 120 mL serum vials with working volume of 100 mL. Food to microorganism (F/M) ratio of 0.3 was used with COD and VSS concentration of 2 and 6 g/L, respectively. To control initial COD concentration at 2 g/L, the wastewaters were adjusted with basal medium which consisted of (NH)_2_SO_4_ 132 mg/L, NaH_2_PO_4_·H_2_O 75.5 mg/L, CaCl_2_·2H_2_O 50 mg/L, MgSO_4_·7H_2_O 90 mg/L, yeast extracts 10 mg/L, and nutrient solution 0.3 mL/L. Nitrogen gas was used to flush vial headspace prior to be closed and sealed with rubber and aluminium caps and then incubated at 37°C. Biogas production and composition were analyzed three times per week until constant biogas accumulation. CH_4_ production rate was calculated by dividing methane production (mL) by the time of maximum methane production (day), while CH_4_ yield was calculated by dividing cumulative CH_4_ volume (mL) with initial COD_added_.

Only six combinations between different starter seeds and wastewaters were selected from 25 factorial experiment design based on the result of maximum CH_4_ production time for further step. Those experiments were conducted in a glass digester with working volume of 1 L. 5 g VSS/L of starter seed was inoculated in these digesters and organic loading rate (OLR) was stepwise increased from 0.5 to 2.0 kg COD/m^3^·d during start-up period based on the COD concentration of wastewaters. The digesters were semicontinuously operated at HRT of 5 days under ambient temperature. Digester performances, such as COD removal, pH, TVA/alkalinity, and CH_4_ production, were investigated. Microbial activity and community were also analyzed at the end of start-up period.

### 2.4. Analytical Methods

#### 2.4.1. Wastewater Characteristics

All raw wastewaters were stored at 4°C before being used in this study. The characteristics of wastewaters, such as pH, alkalinity, TVA, COD, TS, VS, ash, oil and grease, TKN, and protein, were analyzed based on APHA Standard Methods [9]. The carbohydrate concentration was determined by difference between 100 and sum of the percentages of moisture, crude protein, lipid, and ash [10].

#### 2.4.2. Reactor Performance

pH, alkalinity, and TVA were analyzed daily according to standard method [9]. Soluble and total COD were analyzed every 2 days. Biomass concentration, VSS, and TSS were analyzed at initial and final of treatment [9]. Biogas production was analyzed by water replacement method and its composition was analyzed by gas chromatography (GC) 14B with thermal conductivity detector (Shimadzu, Japan).

#### 2.4.3. Microbial Activity

Specific glucose utilization (SGU) represented the activity of starter seed to degrade glucose. 3 g VSS/L of starter seed was inoculated in 120 mL serum vial with glucose (1 g/L) as main substrate to achieve F/M ratio of 0.3. N_2_ gas (99.99%) was flushed to vial headspace to remove oxygen prior to be closed and sealed with rubber and aluminium caps. The vial was incubated in 37°C incubator. Sampling was conducted every 2 hours for 24 hours and glucose concentration was analyzed by dinitrosalicylic acid (DNS) method [11]. Maximum slope of glucose degradation was calculated as maximum SGU activity. SGU was expressed as an amount of COD-glucose which was degraded by one-gram VSS of microorganism per hour (g COD-glucose/g VSS·h).

Specific methanogenic assay (SMA) represented the activity of acetoclastic methanogen in sample [12]. Sodium acetate of 1 g/L was used as main substrate. 3 g VSS/L was also inoculated into 120 mL serum vial for F/M ratio of 0.3. N_2_ gas was flushed for vial headspace. The vial will be incubated under 37°C. Biogas production and composition, as a result from acetate degradation, were daily analyzed until constant accumulation of biogas production. Maximum slope of methane production was considered as SMA value in which mL CH_4_ was converted as g COD-CH_4_. SMA value was defined as amount of g COD which was degraded by one-gram VSS of microorganism per day (g COD-CH_4_/g VSS·d).

#### 2.4.4. Microbial Community

Total genomic DNA was extracted from samples according to the method of [13]. The bacterial 16S rRNA gene was amplified by PCR with the bacterial primer EUB8F and U1492R in the first round and the specific primer set 338GC-F and 518R in the second round [14]. The archaeal 16S rRNA gene was amplified by PCR with the archaeal primer A20F and U1492R in the first round and the specific primer set 344GC-F/522R in the second round [14, 15].

The 200 bp PCR fragments were analyzed by DGGE on the DGGE-2000 system apparatus (CBS Scientific Co. Inc., USA). The equal volumes of PCR products were run on a DGGE with 8% polyacrylamide gels in 1x TAE (Tris-acetate-EDTA) buffer. The gradients were created by the addition of 0–80% denaturant (5.6 M urea and 40% v/v formamide) into polyacrylamide [16]. A 45–70% denaturing gradient was used for the domain of* Eubacteria*, while 40–55% denaturing gradient was used for the domain of* Archaea*. Electrophoresis was performed at 200 V for 5 h and at a constant temperature of 60°C. After electrophoresis, the gels were stained with SYBR Gold nucleic acid stain (Molecular Probes, USA) for 15 minutes. The image was then visualized on UV transilluminator and was captured using Biovision CN 1000/26 M (Vilber Lourmat, France).

Most of the bands were excised from the gel by using Gel Cutting Tips (Cleaver Scientific, England) and reamplified with the primer 338GC-F/518R and 344GC-F/522R for the domain of* Eubacteria *and* Archaea*, respectively. The PCR products were purified using the Gel/PCR DNA fragments extraction kit (Geneaid, Taiwan) according to the manufacturer's instruction. The purified PCR products were sequenced using the 1st BASE Laboratories Sdn Bhd (Malaysia). Sequences were initially compared to known 16S rRNA sequences in the GenBank™ database using the BLAST to locate nearly exact matches in the GenBank database [17].

## 3. Results and Discussion

### 3.1. Selection for Efficient Combination of Exogenous Starter Seed and Various Wastewaters

Lag time for CH_4_ production, CH_4_ production rate, and ultimate CH_4_ production time were considered as main selection parameters.  Table 3 shows that each experiment of combination starter seeds and wastewater was able to produce CH_4_ with different lag time and production rate. The combination between wastewater from swine farm (SWw) and various starter seeds showed the slowest ultimate CH_4_ production time and rate. sCOD/tCOD ratio of SWw was the lowest, approximately 11%, compared to RBw, CSw, Pow, and SMw as approximately 98, 56, 51, and 41%, respectively (Table 2). Low sCOD/tCOD ratio indicated high insoluble particles and solids fractions which was difficult to be degraded by microorganism. Contrary, highest sCOD/tCOD ratio in RBw resulted in higher CH_4_ production rates as approximately 12.37, 8.95, 8.32, 7.08, and 5.91 mL CH_4_/g·VSS·d with starter seed combinations of CSs, POs, SWs, SMs, and RBs, respectively.

Initial SMA of starter seeds also affected microbial activity to degrade different wastewater characteristics. Based on preliminary results, SMA values of SWs, POs, CSs, SMs, and RBs were approximately 0.11, 0.10, 0.09, 0.07, and 0.01 g COD-CH_4_/g VSS·d, respectively. RBs had the lowest SMA value which affected its performance on wastewater degradation. In the easiest degradable and indigenous concentrated rubber wastewater (RBw), RBs also showed the lowest ultimate CH_4_ production times and rates compared to that of another starter seeds. RBs had longer lag phase for CH_4_ production, approximately 5–10 days, which indicated that microorganism in this starter seed needed time to adapt on different wastewaters due to its low activity. The fastest or highest of CH_4_ production slopes were observed in wastewaters combination with SWs and CSs followed by POs, SMs, and RBs.

The efficacies of 5 different various starter seeds with different wastewaters considering ultimate CH_4_ production time, rate, and lag time are shown in Table 3. CH_4_ production rates varied between 1.71 and 12.37 mL CH_4_/g VSS·d, in which most RBw combination showed highest ultimate CH_4_ production times and rates. These parameters were important for determining the application of starter seed and wastewater combinations in next step with stepwise increase of OLR operation. The fastest ultimate CH_4_ production time in each representative starter seeds were selected. Six conditions of combinations starter seed and wastewater were selected for next phase regarding on ultimate CH_4_ production time in order to deeply observation the efficacies of starter seed application for exogenous wastewaters. Combinations of CSsRBw, POsRBw, SMsPOw, RBsSWw, and SWsCSw were selected based on ultimate CH_4_ production time for CSs, POs, SMs, RBs, and SWs, respectively, while CSsSWw was also selected concerning on ultimate CH_4_ production time for combination using swine wastewater (SWw).

### 3.2. Application of Selected Combination of Different Starter Seeds and Wastewater for Start-Up Period

**Figure 1:**
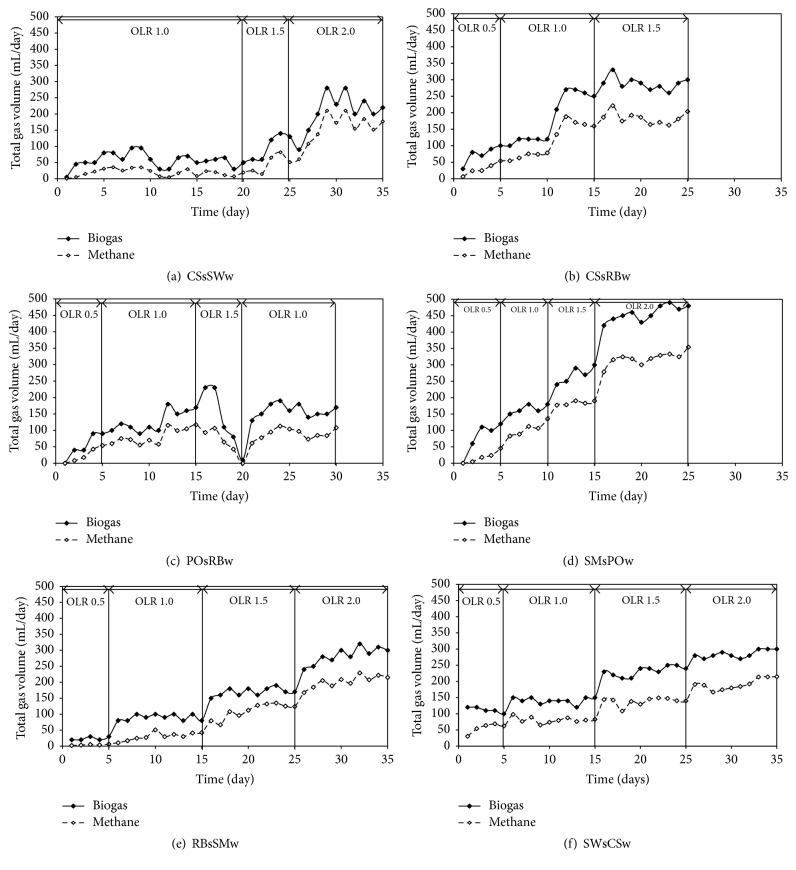
Biogas and methane production of six conditions, CSsSWw (a), CSsRBw (b), POsRBw (c), SMsPOw (d), RBsSMw (e), and SWsCSw (f).

Six selected combinations of different starter seed and wastewater, that is, CSsRBw, POsRBw, SMsPOw, RBsSWw, SWsCSw, and CSsSWw, were semicontinuously operated in larger reactor with stepwise increase of OLR 0.5–2.0 kg COD/m^3^·d with HRT of 5 days.  Figure 1 shows the biogas and methane production during start-up period in each combination. Only four combinations of different starter seed and wastewater, that is, RBsSMw, SMsPOw, SWsCSw, and CSsSWw, could achieve targeted OLR of 2.0 kg COD/m^3^·d as shown in Table 4, while CSsRBw treatment can only be operated at maximum OLR of 1.5 kg COD/m^3^·d and POsRBw treatment was failed at step increasing to OLR 1.5 kg COD/m^3^·d, and however it can recover back to OLR of 1.0 kg COD/m^3^·d until the end of operation. However various COD removals, methane yield, and production were observed in all reactor. Highest CH_4_ production and yield were observed in SMsPOw as 320 mL/d and 160 L CH_4_/kg COD_added_, respectively, which were followed with those in RBsSMw, SWsCSw, and CSsSWw. The lowest CH_4_ production and yield were found at the combination of POsRBw as approximately 90 mL CH_4_/d and 90 L CH_4_/kg COD_added_, respectively.

Based on the initial SMA results of starter seeds for these combinations, the activity of SWs, POs, and CSs was the higher compared to that of SMs and RBs as shown in Table 4, while rubber wastewater (RBw) was the easiest degradable wastewater (based on ultimate methane production time) due to its high sCOD/tCOD ratio. However, the results of start-up operation for the combination between high active seeds and easy-degradable wastewater, such as POsRBw and CSsRBw, could not reach the targeted OLR of 2.0 kg COD/m^3^·d and resulting poor performance in terms of COD removals, CH_4_ productions, and yields. On the contrary, high COD removals, CH_4_ productions, and yields were observed in SMsPOw and RBsSMw (combination between less active seeds and medium sCOD/tCOD ratio wastewater).

Food to microorganism (F/M) ratio applied in preliminary vial test and start-up of semicontinuous reactor were different. Fixed F/M ratio of 0.3 was used for vial test and stepwise F/M ratios from 0.5 to 2.0 were applied for start-up operation of semicontinuous reactor. As a result, easily degraded wastewater, such as RBw, was rapidly degraded by high active seeds which led to organic acids accumulation in reactor. This assumption was supported by TVA/alkalinity ratio in reactor of CSsRBw and POsRBw which were higher, approximately 0.4–0.6, compared to that in other combinations. The balance between acidogens and methanogens in these reactors may also affect their performances. It can be noticed from the result of Table 4. Moreover, the wastewater generated from concentrated rubber latex industry contains high organic, ammonia nitrogen, and sulfate concentrations. Ammonia in large amount is used as preservative agent for rubber latex and sulfuric acid is needed for recovering rubber particles in waste stream [18]. These high ammonia and sulfate adversely affect the microbial activity in anaerobic digestion system, especially methanogens as the most sensitive microorganism [19].

The specific glucose utilization, representative for activity of acidogens, in these reactors sharply increased, while the activity of acetoclastic methanogens (SMA) decreased as approximately 38, 15, and 36%, respectively. High active acidogens could lead to fast acids production and low pH in the system which affected the activity of acetoclastic methanogens. However, glucose utilization in SMsPOw and RBsSMw was very low and the increase of activity of acetoclastic methanogens in these reactors was approximately 350 and 150%, respectively. Organic acids from acidogenic activity were expected to be used directly by methanogens to produce methane which resulted in less acids accumulation and low TVA/alkalinity ratio inside reactors. From these results, it can be stated that the parameter of F/M ratio, the characteristics of seeds, and wastewaters must be notably considered for reactor operation with combination of various starter seeds and wastewaters.

### 3.3. Microbial Community Based on DGGE Profiles

Tables 5 and 6 summarize the observed bands and their related microorganism along with the percent of similarities for the bacterial and archaeal communities in initial seed starters and combination of between seed starters and wastewater at the end of start-up period. In anaerobic digestion,* Eubacteria* is distinguished into three main groups, namely, hydrolytic, acidogenic, and acetogenic groups. Those three main microbial groups were observed in all initial starter seeds with different dominant microorganism. Starter seed from swine manure WWTP consisted of the most* Eubacteria* compared to other starter seeds. 4 out of 5 hydrolytic, 3 out of 5 acidogenic, and 5 out of 8 acetogenic microorganisms were detected from SWs, while RBs showed the least detected bacteria (2 out of 5 hydrolytic, 1 out of 5 acidogenic, and 2 out of 8 acetogenic bacteria).

Acetogenic bacteria have important role for oxidizing products from acidogenesis and providing appropriate substrate for methanogens. Therefore, acetogens (H_2_ producers) syntrophically collaborate with methanogens (H_2_ consumers) [5]. 5 dominant bands of acetogens were detected in SWs, more than other starter seeds.* Syntrophus *sp. and* Smithella propionica *strain LYP were observed in all starter seeds. These microorganisms were considered as acetogens in which* Smithella propionica* strain LYP utilized propionate to produce acetate and hydrogen and* Syntrophus *sp. syntrophically cooperate with hydrogenotrophic methanogens [20, 21]. Several sulfate reducing bacteria, such as* Sulfuricurvum kujiense *strain DSM 16994,* Syntrophus gentianae *strain HQgoel,* Desulfococcus biacutus,* and* Shewanella amazonensis*, and one of nitrate reducing bacteria (*Nitratiruptor *sp.) were also detected in SWs. These dominant bacteria found in SWs were affected by the characteristics of swine manure wastewater which contained high protein, carbohydrate, lipid, and total nitrogen as shown in Table 1. High SRB found in SWs was probably caused by high sulfur concentration in swine manure wastewater [22, 23].

**Figure 2:**
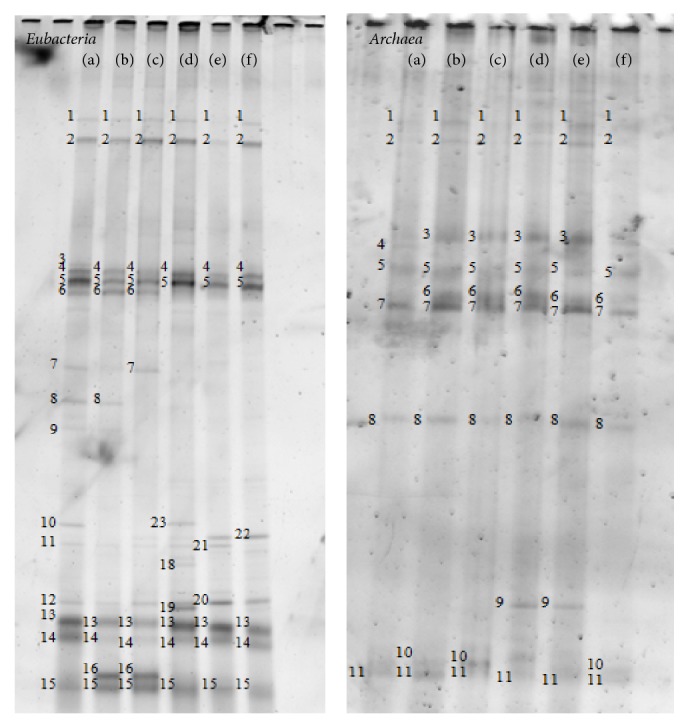
DGGE pattern of* Eubacteria *and* Archaea *groups from various combinations, CSsSWw (a), CSsRBw (b), POsRBw (c), RBsSMw (d), SMsPOw (e), and SWsCSw (f).

*Eubacteria* in the combination between starter seeds and wastewaters during start-up period were also investigated (Figure 2 and Table 5). The combination of SMsPOw, RBsSMw, and SWsCSw, which showed good performance until OLR 2.0 kg COD/m^3^·d, has few bacteria. Two hydrolytic, three acidogenic, and one acetogenic microorganism were detected in the highest performance combination of SMsPOw, while combination of POsRBw, the lowest performance, has 8 dominant bacteria as 2 bacteria of each hydrolytic, acidogenic, and acetogenic microorganism and SRB.* Arcobacter cryaerophilus* strain A 169/B (band 12) and* Sunxiuqinia faeciviva *strain JAM-BA0302 (band 5) were detected in all samples as carbohydrate- and protein-degrading microorganism, respectively [24, 25]. More protein-degrading microorganism,* Polaribacter porphyrae *strain LNM-20 (band 7), was detected in combination of CSsSWw, CSsRBw, and POsRBw. Protein concentration in wastewaters of SWw and RBw was higher compared to other wastewaters, as approximately 40.47 and 10.98 g/L, respectively. It is clear that wastewaters may contain their indigenous microorganism. Acetogenic (amino acid degrader),* Candidatus Cloacamonas acidaminovorans *strain Evry (band 8), was also detected in combinations of CSsSWw and CSsRBw. More SRB also detected in combination starter seeds with swine manure wastewater.

Three main groups of methanogens, namely, methylotrophic, hydrogenotrophic, and acetoclastic methanogens, were observed at both sample conditions as shown in Table 6.* Methanomethylovorans hollandica *strain DSM 15978 was detected at band 8 (initial starter seed) and band 7 (combination during start-up) in all samples. This microorganism is isolated from freshwater sediment and obligately methylotrophic methanogen since it utilizes only methanol, methylamines, methanethiol, and dimethyl sulfide [26]. Another methylotrophic methanogen,* Methanosarcina acetivorans *strain C2A, was observed as band 3 in some combination treatment (CSsRBw, POsRBw, RBsSMw, and SMsPOw). Carbon monoxide is utilized by this microorganism to produce methane via a pathway that involves H_2_ as an intermediate [27]. Three hydrogenotrophic methanogens were observed in combination treatments such as* Methanolinea mesophila *strain TNR (band 4 in CSsSWw),* Methanolinea tarda *strain NOBI-1 (band 5 in all samples), and* Methanoregula formicica *strain SMSP (band 6 in CSsRBw, POsRBw, RBsSMw, SMsPOw, and SWsCSw), while only one hydrogenotrophic methanogen (*Methanoregula formicica *strain SMSP) was detected in initial starter seeds of CSs and SWs as band 9. These hydrogenotrophic methanogens are mesophilic and utilize H_2_/CO_2_ and formate to produce methane [28–30].* Methanosaeta concilii *GP6, an acetoclastic methanogen, was detected in all initial starter seeds (band 2) and in all combination treatments (bands 1, 2, 8, and 11).* Methanosarcina mazei *Go1 was only detected in combination treatments of CSsRBw and POsRBw at band 10. Another acetoclastic methanogens, that is,* Methanosaeta harundinacea, *was only detected in initial all starter seeds. This microorganism only grows in very high acetate concentration (>100 mM) as exclusive substrate [31]. It can be assumed that the absence of this microorganism in combination treatment was probably caused by low acetate production during start-up period of this treatment. Acetoclastic methanogens, that is,* Methanosarcina* and* Methanosaeta, *contribute approximately two-thirds of methane production from acetate.* Methanosaeta* spp. are widely distributed in natural environment and their filamentous cell trigger sludge granulation [31]. Another uncultured* Archaea* were also detected in initial seed starters as shown in Table 6. It seems that wastewaters used as combination treatment may contain its indigenous archaeal microorganism since more methanogens were detected at this sample.

## 4. Conclusions

The efficacies of exogenous starter seeds to treat different wastewater were investigated in this study. All of exogenous anaerobic starter seeds were able to produce CH_4_ from different sources of wastewater, although the different CH_4_ production rates and lag phases were observed. The effectiveness of using different exogenous anaerobic starter seeds to treat different wastewater types was not only determined by the quality of starter seeds and wastewaters but also considered by the F/M ratio and microbial community inside starter seeds. It was shown by the results above in which the combination between high active starter seeds and easy-degradable wastewaters (high sCOD/tCOD ratio), such as CSsRBw and POsRBw, could not achieve the targeted OLR of 2 kg COD/m^3^·d. On the contrary, less active starter seeds with medium-degradable wastewater, such as RBsSMw and SMsPOw, showed good performances until reaching the targeted OLR. Therefore, it can be assumed that F/M ratio affected these results via microbial community and their microbial activities. High F/M ratio with easy-degradable wastewater initiated fast microbial degradation (higher SGU) which led to acids accumulation in reactor and decreased methanogens activity (lower SMA). As such, this F/M parameter needs to be considered for starting up new anaerobic reactor with exogenous starter seeds and easy-degradable wastewaters. Based on DGGE profiles,* Smithella propionica *strain LYP and* Syntrophus *sp. were detected in all of initial starter seeds and selected combinations of different starter seeds and wastewaters. For* Archaea* domain, methylotrophic, hydrogenotrophic, and acetoclastic methanogen were detected in all samples which were represented by* Methanomethylovorans hollandica *strain DMS1,* Methanoregula formicica *strain SMSP, and* Methanosaeta concilii *strain GP6, respectively. Propionate utilizer (*Smithella propionica *strain LYP) was assumed to produce acetate and hydrogen for those methanogens under syntrophic relationship.

## Figures and Tables

**Table 1:** The characteristics of various wastewaters used in this study.

Parameter	Unit	RBw	CSw	POw	SWw	SMw
pH	—	4.58	4.58	4.26	7.24	4.06
Alkalinity	mg/L	1,100	400	1,100	13,000	400
Total volatile acid (TVA)	mg/L	3,460	1,100	6,117	11,000	100
Total COD (TCOD)	mg/L	17,200	25,200	86,200	181,100	7,300
Soluble COD (SCOD)	mg/L	16,900	14,200	45,000	19,100	3,000
Total Kjeldahl nitrogen (TKN)	mg/L	1,800	400	1,100	6,500	300
Total solids (TS)	g/L	16.40	19.37	60.05	188.20	4.67
Volatile solids (VS)	g/L	13.82	16.91	50.24	146.9	4.09
Protein (P)	g/L	10.98	2.30	6.78	40.47	1.55
Carbohydrate (C)	g/L	2.84	13.97	29.89	81.30	1.73
Oil & grease (O)	g/L	0	0.63	13.56	16.10	0.82
P : C : O ratio	—	4 : 1 : 0	4 : 22 : 1	1 : 4 : 2	3 : 5 : 1	2 : 2 : 1

**Table 2:** 5 × 5 factorial experiment design of starter seeds and wastewaters.

Starter seed	Wastewater	Abbreviation
Concentrated rubber (RBs)	Concentrated rubber (RBw)	RBsRBw
	Cassava starch (CSw)	RBsCSw
	Palm oil mill (POw)	RBsPOw
	Swine manure (SWw)	RBsSWw
	Soymilk processing (SMw)	RBsSMw

Cassava starch (CSs)	Concentrated rubber (RBw)	CSsRBw
	Cassava starch (CSw)	CSsCSw
	Palm oil mill (POw)	CSsPOw
	Swine manure (SWw)	CSsSWw
	Soymilk processing (SMw)	CSsSMw

Palm oil mill (POs)	Concentrated rubber (RBw)	POsRBw
	Cassava starch (CSw)	POsCSw
	Palm oil mill (POw)	POsPOw
	Swine manure (SWw)	POsSWw
	Soymilk processing (SMw)	POsSMw

Swine manure (SWs)	Concentrated rubber (RBw)	SWsRBw
	Cassava starch (CSw)	SWsCSw
	Palm oil mill (POw)	SWsPOw
	Swine manure (SWw)	SWsSWw
	Soymilk processing (SMw)	SWsSMw

Soymilk processing (SMs)	Concentrated rubber (RBw)	SMsRBw
	Cassava starch (CSw)	SMsCSw
	Palm oil mill (POw)	SMsPOw
	Swine manure (SWw)	SMsSWw
	Soymilk processing (SMw)	SMsSMw

**Table 3:** Order of 25 factorial experiments based on rate and ultimate time of methane production.

Number	Experiment	Ultimate CH_4_ production time (day)	CH_4_ production rate (mL CH_4_/g VSS·d)	Lag time (days)
1	CSsRBw^*∗*^	7	12.37	0
2	SWsRBw	8	8.32	0
3	POsRBw^*∗*^	9	8.95	0
4	SMsPOw^*∗*^	12	4.51	0
5	CSsPOw	14	7.36	0
6	SMsRBw	14	7.08	0
7	SMsSMw	14	6.94	0
8	CSsCSw	14	6.21	0
9	RBsRBw	17	5.91	0
10	POsPOw	17	4.77	0
11	POsCSw	17	4.35	0
12	CSsSMw	20	6.38	0
13	RBsPOw	20	5.37	0
14	RBsSMw^*∗*^	20	5.03	0
15	CSsSWw^*∗*^	20	3.37	0
16	SMsCSw	22	4.78	6
17	POsCSw	22	4.13	3
18	POsSWw	22	2.38	0
19	SWsCSw^*∗*^	24	3.86	0
20	SWsPOw	24	3.74	0
21	SWsSMw	24	2.69	0
22	RBsCSw	30	3.92	4
23	RBsSWw	30	3.01	0
24	SWsSWw	30	2.65	0
25	SMsSWw	32	1.71	0

^*∗*^Selected combinations of different starter seed and wastewater for next phase.

**Table 4:** Performances of six selected combinations of different starter seed and wastewater.

Parameter	CSsRBw	POsRBw	RBsSMw	SMsPOw	SWsCSw	CSsSWw
SMA_initial_ (g COD-CH_4_/g VSS·d)	0.13	0.11	0.02	0.06	0.08	0.13

SMA_final_ (g COD CH_4_/g VSS·d)	0.11	0.07	0.09	0.12	0.09	0.08

SGU_initial_ (g COD/g VSS·h)	0.26	0.29	0.18	0.38	0.38	0.26

SGU_final_ (g COD/g VSS·h)	0.37	0.39	0.20	0.29	0.41	0.43

Final OLR (kg COD/m^3^·d)	1.5	1.0 (recovery)	2.0	2.0	2.0	2.0

TVA/alkalinity ratio	0.4–0.6	0.4–0.6	0.3–0.5	0.3–0.5	0.5–0.6	0.4–0.5

Av. sCOD removal (%)	94	74	96	75	93	87

Av. tCOD removal (%)	85	55	76	57	79	67

Av. biogas production (mL/d)	289	160	284	457	285	209

Av. CH_4_ production (mL/d)	185	90	203	320	192	157

CH_4_ yield (L/kg COD_added_)	123	90	101	160	96	78

OLR achieved	Not reach OLR2, max. OLR 1.5	Fail at OLR 1.5, at recovered OLR 1.0	OLR 2 at day 35	OLR 2 at day 25	OLR 2 at day 35	OLR 2 at day 35

**Table 5:** DGGE profiles of *Eubacteria *domain in initial starter seeds and six selected combinations.

Bacteria group in AD	Affiliation	Similarity (%)	Accession number	Initial starter seeds	Start-up period					
					CSsSWw	CSsRBw	POsRBw	RBsSMw	SMsPOw	SWsCSw
Hydrolytic	*Polaribacter porphyrae* strain LNM-20	81	NR_114321.1		+ (7)	+ (7)	+ (7)			
	*Arcobacter cryaerophilus* strain A 169/B	96	NR_025905.1		+ (12)	+ (12, 17)	+ (12, 17)	+ (12)	+ (12)	+ (12)
	*Sunxiuqinia faeciviva* strain JAM-BA0302	77	NR_108114.1		+ (13)	+ (13)	+ (13)	+ (13)	+ (13)	+ (13)
	*Flavobacterium psychrophilum*	86	NR_074630.1	CSs (6)						
	*Acanthopleuribacter pedis*	95	NR_041599.1	SWs (8)						
	*Microbulbifer donghaiensis*	78	NR_044478.1	RBs, POs, SWs, SMs (11)						
	*Alkanindiges illinoisensis*	87	NR_025254.1	CSs, POs, SWs, SMs (12)						
	*Gelidibacter algens*	89	NR_026026.1	RBs, SWs (20)						

Acidogenic	*Roseateles terrae* strain CCUG 52222	98	NR_042616.1					+ (18)		
	*Reichenbachiella agariperforans* strain NBRC 16625	80	NR_113854.1		+ (5)	+ (5)	+ (5)	+ (5)	+ (5)	+ (5)
	*Acinetobacter johnsonii* strain Mannheim 3865/60	99	NR_044975.1					+ (20)	+ (20, 21)	
	*Maricaulis maris MCS10* strain MCS10	83	NR_074160.1						+ (22)	+ (22)
	*Acinetobacter sp.*	94	AF189693.1	SWs, SMs (10, 13)						
	Uncultured* Bacteroidetes bacterium*	87	JQ012284.1	RBs, CSs, SWs (17)						
	*Clostridium perfringens*	91	NR_074482.1	SWs, SMs (18)						
	Uncultured* Clostridium sp.*	100	KF003155.1	POs (27)						
	*Clostridium papyrosolvens*	83	NR_026102.1	SMs (28)						

Acetogenic	*Smithella propionica* strain LYP	93	NR_024989.1	All (2)	+ (2, 4)	+ (2, 4)	+ (2, 4)	+ (2, 4)	+ (2, 4)	+ (2, 4)
	*Candidatus Cloacamonas acidaminovorans* strain Evry	89	NR_102986.1		+ (8)	+ (8)		+ (23)		
	*Bizionia hallyeonensis* strain Ty7	92	NR_109525.1		+ (10)					
	*Marinobacter goseongensis* strain En6	79	NR_044340.1			+ (16)	+ (16)			
	*Syntrophus sp.*	92	AJ133796.1	All (1)						
	Uncultured* Syntrophaceae bacterium*	99	GQ242556.1	RBs, CSs, SWs (7, 18)						
	*Dehalococcoides mccartyi*	87	NR_102515.1	SWs, SMs (14)						
	*Dehalogenimonas lykanthroporepellens*	90	NR_074337.1	POs (16)						
	*Syntrophus aciditrophicus*	93	NR_102776.1	CSs (22)						
	*Sedimentibacter saalensis *	89	NR_025498.1	CSs, POs, SMs (23)						
	*Geobacter uraniireducens*	78	NR_074940.1	SWs (25)						
	*Syntrophaceae bacterium *enrichment culture	97	JX473531.1	SWs (30)						

Others	*Nitrosomonas communis* strain NM2	73	NR_104887.1		+ (1)	+ (1)	+ (1)	+ (1)	+ (1)	+ (1)
	*Sulfuricurvum kujiense* strain DSM 16994	91	NR_074398.1		+ (6)	+ (6)	+ (6)			
	*Kordia algicida* strain NBRC 100336	86	NR_113886.1		+ (14)	+ (14)	+ (14)	+ (14)	+ (14)	+ (14)
	*Acinetobacter radioresistens* strain NBRC 102413	89	NR_114074.1				+ (15)			
	*Acidovorax defluvii* strain BSB411	98	NR_026506.1				+ (19)			
	*Sulfurospirillum halorespirans* strain PCE-M2	94	NR_028771.1		+ (11)					
	*Geitlerinema sp.*	77	NR_102448.1	CSs (9)						
	*Bacterium *enrichment culture	99	JF947012.1	RBs, POs, SMs (3)						
	*Syntrophus gentianae* strain HQgoe1	87	NR_029295.1	CSs, SWs, SMs (4)	+ (3)	+ (3)	+ (3)	+ (3)	+ (3)	+ (3)
	*Desulfococcus biacutus*	79	NR_025406.1	SWs (15)	+ (9)	+ (9)				
	*Nitratiruptor sp.*	89	NR_074874.1	CSs, SWs, SMs (21)						
	*Uncultured bacterium*	96	JQ799939.1	All (5, 24)						
	*Thermosynechococcus elongatus*	79	NR_074328.1	SWs (26)						
	*Shewanella amazonensis*	78	NR_074842.1	POs, SWs (29)						

(Number) = band in DGGE analysis and + (number) = the presence of microorganism in DGGE band.

**Table 6:** DGGE profiles of *Archaea *domain in initial starter seeds and six selected combinations.

Archaea group	Closest match	Similarity (%)	Accession number	Initial starter seeds	Start-up period					
					CSsSWw	CSsRBw	POsRBw	RBsSMw	SMsPOw	SWsCSw
Methylotrophic methanogens	*Methanosarcina acetivorans* strain C2A	80	NR_074110.1			+ (3)	+ (3)	+ (3)	+ (3)	
	*Methanomethylovorans hollandica *strain DSM 15978	93	NR_102454.1	All (band 8)	+ (7)	+ (7)	+ (7)	+ (7)	+ (7)	+ (7)
	*Methanomethylovorans hollandica *strain DMS1	99	NR_028174.1					+ (9)	+ (9)	

Hydrogenotrophic methanogens	*Methanolinea mesophila *strain TNR	86	NR_112799.1		+ (4)					
	*Methanolinea tarda *strain NOBI-1	86	NR_028163.1		+ (5)	+ (5)	+ (5)	+ (5)	+ (5)	+ (5)
	*Methanoregula formicica *strain SMSP	82	NR_102441.1	CSs, SWs (band 9)		+ (6)	+ (6)	+ (6)	+ (6)	+ (6)

Acetoclastic methanogens	*Methanosaeta concilii *strain GP6	89	NR_102903.1	All (band 2)	+ (1, 2, 8, 11)	+ (1, 2, 8, 11)	+ (1, 2, 8, 11)	+ (1, 2, 8, 11)	+ (1, 2, 8, 11)	+ (1, 2, 8, 11)
	*Methanosaeta concilii *strain Opfikon	94	NR_028242.1	CSs (band 3)						
	*Methanosarcina mazei *Go1	98	NR_074221.1			+ (10)	+ (10)			
	*Methanosaeta harundinacea*	99	NR_102896.1	All (band 11)						

Another *Archaea*	Uncultured *methanogenic archaeon*	88	HQ141845.1	CSs, SWs (band 1)						
	*Methanogenic prokaryote *enrichment culture	99	KC821320.1	All (band 4)						
	Uncultured *archaeon *clone	91	EF639564.1	All (band 5)						
	Uncultured *Methanobacteriaceae archaeon*	92	GU982677.1	All (band 6)						
	Uncultured *Methanosaeta *sp.	99	AY454766.1	POs (band 7)						
	Uncultured *Methanosaeta *sp.	99	AY454766.1	SMs (band 10)						

(Number) = band in DGGE analysis and + (number) = the presence of microorganism in DGGE band.
